# The Patterning of Exposures Among Aging Populations in Eight Nations

**DOI:** 10.1093/geroni/igaf122.043

**Published:** 2025-12-31

**Authors:** Nia Clements

**Affiliations:** University of Michigan School of Public Health, Ann Arbor, Michigan, United States

## Abstract

This presentation will showcase the variability in population-weighted exposome measures for Brazil, Chile, England, India, Ireland, Northern Ireland, and the United States using harmonized data from Gateway. We used the global exposure measures derived from satellite data, empirical spatiotemporal prediction models, and physics-based deterministic models to estimate exposures at population density weighted locations in each country. We then evaluated patterns within and between countries across space and time. More pronounced differences were observed between countries for several key ambient air pollutants such as particulate matter and ozone whereas the variation within countries was larger for measures of natural spaces like greenspace and nitrogen dioxide as a traffic-related air pollutant. For instance, median particulate matter levels were nearly seven times higher in India than in the United States (52.8 ug/m3 versus 7.6 ug/m3 respectively). Although temporal trends and correlations between exposures were often consistent across countries, we will highlight some unique patterns that emerged, such as how from 1990 to 2017, ozone in India increased by 0.52 ppb per year on average whereas ozone in Mexico decreased by 0.60 ppb per year on average. Furthermore, from 2017 to 2018 alone, there was a 7.6% decrease in greenspace in Chile while greenspace levels were relatively constant in other countries evaluated. The talk will conclude with implications of similarities and differences in exposures patterns for research on environmental predictors of accelerated aging, such as opportunities for change-on-change modeling and the influence of place-based confounding by different exposures.

